# An Isolated Bee Sting Involving Multiple Cranial Nerves

**DOI:** 10.1155/2013/920928

**Published:** 2013-07-18

**Authors:** Hassan Motamed, Arash Forouzan, Fatemeh Rasooli, Alireza Majidi, Mohammadreza Maleki Verki

**Affiliations:** ^1^Emergency Department, Ahvaz Jundishapur University of Medical Sciences, Ahvaz, Iran; ^2^Emergency Department, Shahid Beheshti University of Medical Sciences, Tehran, Iran

## Abstract

Hymenoptera stings are self-limiting events or due to allergic reactions. Sometimes envenomation with Hymenoptera can cause rare complications such as acute encephalopathy, peripheral neuritis, acute renal failure, nephrotic syndrome, silent myocardial infarction, rhabdomyolysis, conjunctivitis, corneal infiltration, lens subluxation, and optic neuropathy. The mechanism of peripheral nervous system damage is not clearly known. In our studied case after bee sting on face between the eyebrows with little erythema and 1 × 1 cm in size, bilateral blindness developed and gradually improved. Lateral movement of eyes was restricted with no pain. Involvement of cranial nerves including II, V, and VI was found. With conservative therapy after a year significant improvement has been achieved.

## 1. Introduction

The Hymenoptera are one of the four groups of insects [1]. The majority of Hymenoptera stings are self-limiting events and resolve in minutes to hours without treatment [2]. Among animals that produce venom and effect humans, bee stings have a high mortality rate [3]. They can cause severe adverse effects, such as anaphylactic reactions, cardiovascular collapse, and death [1]. One rare complication of bee stings is optic neuritis which is not fully understood [4]. The following case presents the involvement of several cranial nerves including II, V, and VI.

## 2. Case Presentation

**Figure 1:**
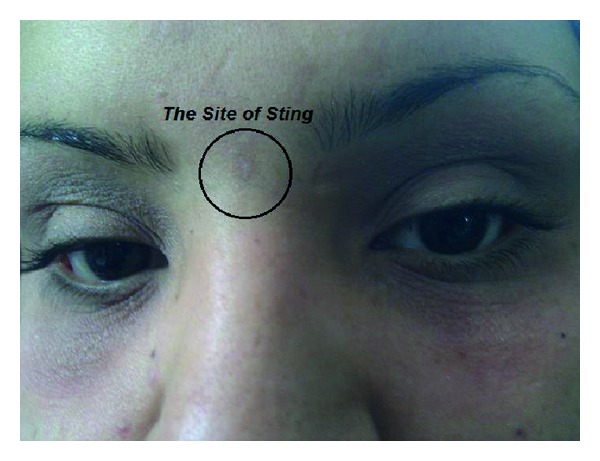
The site of sting in the patient with involved multiple cranial nerves 36 hours after incident.

 A 23-year-old woman suffering from bilateral blindness without previous similar history was referred to the emergency department. Her symptoms have been started 36 hours ago and gradually have been improved. Her past medical history did not show any specific disease or use of medication. On examination she was alert and conscious with normal range of vital signs. The site of sting was seen on the face between the eyebrows with little erythema and 1 × 1 cm in size (Figure 1). There were edema and erythema on the superior and inferior eyelids without any tenderness. The patient was admitted to the neurology ward for further assessment. In the following examinations pupils were of normal size and corneal reflexes were intact. Lateral movement of eyes was restricted with no pain. Facial sensory had declined in levels of V1, V2, and V3 bilaterally. Vision of the right eye was NLP (no light perception) and the left eye was HN (hand motion). Funduscopic examination revealed sharp optic disk margins. There was no evidence of central and peripheral facial nerve palsy. Auditory tests were established that resulted in no abnormal findings. Deep tendon reflexes were normal. Grade of muscles force in all limbs was 5/5. ECG showed a normal sinus rhythm. Cerebellar tests were normal. Cranial and orbital computed tomography scan revealed normal findings. Other laboratory tests were normal.

## 3. Discussion

Medically the top list groups of Hymenoptera are the Apoidea (bees), Vespoidea (wasps, hornets, and yellow jackets), and Formicidae (ants) [2]. Hymenoptera can deliver between 100 ng (fire ants) and 50 ng (bees) of venom [5]. In general, definitive identification of the offending insect is unnecessary, because signs and symptoms of envenomation are similar for all species of Hymenoptera. Sometimes envenomation with Hymenoptera can cause death in nonallergic individuals [2]. About 40 deaths a year by Hymenoptera stings are reported [5]. Most deaths are related to immediate hypersensitivity reactions, causing anaphylaxis. These anaphylactic reactions are not dose dependent [2]. The clinically Bee stings consist of anaphylaxis or poisoning [3]. Sometimes there are unusual or unexpected reactions related to insect stings that include acute encephalopathy, peripheral neuritis, acute renal failure, nephrotic syndrome, silent myocardial infarction, rhabdomyolysis, conjunctivitis, corneal infiltration, lens subluxation, and optic neuropathy [6]. The mechanism of peripheral nervous system damage is not clearly known [7]. Because of rapid progress of life-threatening anaphylactic reactions, close monitoring is necessary [2]. In uncomplicated cases (stings) conservative therapy, (antihistamines, ice or cool compresses, topical lidocaine, or corticosteroid lotions) was used [2]. In some studies early treatment with corticosteroids in optic neuritis caused by bee sting was effective [8].

In our studied case, conservative therapy leads to significant improvement in initial symptoms within the first year after attack. After one year the eyes easily move in all directions. Disturbed facial sense returned to normal state. Vision of the right eye completely recovered, but visual acuity in the left eye was 6/10. Optic disk has sharp margin.

## 4. Conclusions

 Optic neuritis is a rare complication after bee sting. But the chief concern is to limit the acceleration of visual compromise. In uncomplicated cases conservative therapy has been effective. In our case, significant improvement achieved during one year with conservative therapy.
